# What is the likely impact on surgical site infections in Australian hospitals if smoking rates are reduced? A cost analysis

**DOI:** 10.1371/journal.pone.0256424

**Published:** 2021-08-25

**Authors:** Nikki McCaffrey, Michelle Scollo, Emma Dean, Sarah L. White

**Affiliations:** 1 Deakin Health Economics, School of Health & Social Development, Centre for Population Health Research, Deakin University, Burwood, Victoria, Australia; 2 Quit, Cancer Council Victoria, Melbourne, Victoria, Australia; 3 Centre for Behavioural Research in Cancer, Cancer Council Victoria, Melbourne, Victoria, Australia; 4 Population Health, Alfred Health, Melbourne, Victoria, Australia; University of Malta Faculty of Health Sciences, MALTA

## Abstract

**Introduction:**

Assisting smokers to quit before surgery reduces surgical site infection (SSI) risk. The short-term economic benefits of reducing SSIs by embedding tobacco dependence treatment in Australian hospitals are unknown. Estimated annual number of SSIs prevented, and hospital bed-days (HBD) and costs saved from reducing smoking before surgery are calculated.

**Methods:**

The most recent number of surgical procedures and SSI rates for Australia were sourced. The number of smokers and non-smokers having a SSI were calculated using the UK Royal College of Physicians reported adjusted odds ratio (1.79), and the proportion of SSIs attributable to smoking calculated. The potential impact fraction was used to estimate reductions in SSIs and associated HBDs and costs from reducing the smoking rates among surgical patients from 23.9% to 10% or 5% targets. Uncertainty around the final estimates was calculated using probabilistic sensitivity analysis.

**Results:**

In 2016–17, approximately 40,593 (95% UI 32,543, 50,239) people having a surgical procedure in Australia experienced a SSI leading to 101,888 extra days (95% UI 49,988, 200,822) in hospital. If the smoking rate among surgical patients was reduced to 10%, 3,580 (95% UI 2,312, 5,178) SSIs would be prevented, and 8,985 (95% UI 4,094, 19,153) HBDs and $19.1M (95% UI $7.7M, $42.5M) saved in one year. If the smoking rate was reduced to 5%, 4,867 (95% UI 3,268, 6,867) SSIs would be prevented, and 12,217 (95% UI 5,614, 25,642) HBDs and $26.0M (95% UI $10.8M, $57.0M) would be saved.

**Conclusions:**

The findings suggest achieving smoking rate targets of 10% or 5% would provide substantial short-term health and economic benefits through reductions in SSIs. Embedding tobacco dependence treatment in Australian hospitals would provide value for money by reducing costs and improving clinical quality and safety. A more comprehensive, modelled economic evaluation synthesising the best available evidence is needed to confirm findings.

## Introduction

Despite overwhelming evidence of the health and economic benefits of smoking cessation across multiple jurisdictions and settings [1], best practice tobacco dependence treatment (TDT)—combining multi-session behavioural intervention with approved pharmacotherapy—is not provided routinely in Australian health care [2–4]. Economic analyses are often used to predict the cost and benefits of public health policies, such as the provision of smoking cessation services, on healthcare systems [5, 6]. These analyses typically estimate reductions in the prevalence and incidence of chronic diseases such as coronary heart disease and cancers due to reductions in the smoking rate through quitting and reduced uptake [7, 8]. However, few population level, economic analyses consider the shorter term costs and benefits of smoking cessation, such as improvements in perinatal and neonatal outcomes [9] and reductions in post-operative complications [10].

Smoking increases post-operative complications as it impairs the immune system [11] and increases the risk of surgical site infection (SSI) due to delayed healing from reduced tissue perfusion and impaired inflammatory processes, proliferation of fibroblasts and collagen production [11–13]. People who smoke are at higher risk of wound complications (pooled adjusted odds ratio (OR) 2.27, 95% CI 1.82–2.84), delayed healing and wound separation (pooled adjusted OR 2.07, 95% 1.53–2.81) and SSIs (pooled adjusted OR 1.79, 95% CI 1.57–2.04) compared with non-smokers [13]. Surgical site infections are one of the most common surgical complications [14], and increase the health and economic burden of surgery through greater morbidity and mortality, poorer quality of life, increased use of diagnostic tests and treatment, additional surgery, extended hospital stays and lost productivity [12, 15]. A recent systematic review of the impact of SSIs on healthcare costs across six European countries, reported on average, an extra 15.5 hospital bed-days (HBD) for people who had cardiothoracic surgery and experienced a SSI [15].

In Australia, SSIs occur in approximately 3% of surgical procedures [16, 17]. Preventing and controlling healthcare-associated infections, including SSIs, is one of eight National Safety and Quality Health Service (NSQHS) standards developed by the Australian Commission on Safety and Quality in Health Care to improve the quality of healthcare provision and protect patients [18]. All Australian hospitals must be accredited to these NSQHS Standards. Smoking, unlike some other risk factors for SSIs, such as age, is a modifiable risk factor. A recent study undertaken by the World Health Organization (WHO), the University of Newcastle, Australia and the World Federation of Societies of Anaesthesiologists, demonstrated that smokers who quit approximately four weeks before surgery have a reduced risk of post-surgical complications such as pulmonary complications and SSIs [19]. Every additional week of smoking cessation prior to surgery (beyond the four weeks) improved post-surgical complications by a further 19% [19].

Providing targeted, relevant and accessible information to hospital decision makers, such as hospital administrators, on the benefits of providing TDT could help promote the adoption of evidence-based practice in hospitals [20, 21]. There is limited evidence of the cost-effectiveness of TDT for preventing post-operative complications prior to total joint arthroplasty [22] and abdominal colon surgery [23]. However, contemporary, population estimates of the more immediate health and economic benefits of embedding best practice TDT in hospitals for *all* types of surgery are unknown. Consequently, this analysis aimed to estimate the annual expected number of SSIs prevented, HBDs and costs saved from reducing the 2016 surgical patient smoking rate (23.9%) to targets of 10% or 5% in 2016 to inform decision makers about the value for money of embedding best practice TDT in Australian hospitals.

## Materials and methods

### Structure

Potential annual reductions in HBDs and hospitalisation costs were estimated from publicly available information using an epidemiological approach informed by prevalence data [17], a meta-analysis [10, 13], hospital statistics [24] and modelling data [25]. A 1-year analysis was developed to estimate the impact of reduced smoking on SSIs in Australian public hospitals from a healthcare perspective. The following inputs were used to estimate the annual expected number of SSIs, HBDs and hospitalisation costs: the total number of surgical procedures conducted in Australian public hospitals in one year [24]; the smoking rate in surgical patients [26–28]; the annual prevalence of SSIs [17]; the adjusted odds ratio for surgical patients who smoke experiencing a SSI versus surgical patients who do not smoke [10, 13]; the excess HBDs associated with SSIs [25]; and the average cost per HBD [29].

### Parameters

Table 1 outlines the steps used to estimate the annual population undergoing a surgical procedure in an Australian public hospital. A literature review was conducted to identify the most recently reported government statistics on the number of surgical procedures in public hospitals, the proportion of surgical patients who smoke and the annual prevalence of SSIs in Australia. PubMed Central®, Google and “Tobacco in Australia” [30] were searched using combinations of the following key terms: Australia, hospital, smoking rate, surgery, surgical site infection, hospital-acquired infection, cost and economic. Forward citation tracing of relevant data sources was also conducted using Web of Science^TM^ to identify more recently published pertinent research.

**Table 1:** Estimated annual expected number of surgical site infections in Australian public hospitals.

	Variable	Point estimate	Number	Source
Total number of surgical procedures in public hospitals, 2016–17	A		1,127,574	AIHW [24]
Proportion of surgical patients who smoke (A x B)	B	23.9%	1,127,574 x 23.9% = 269,490	NDSHS [32]
Proportion of surgical patients who don’t smoke (A x C)	C	76.1%	1,127,574 x 76.1% = 858,084	NDSHS [32]
Proportion of surgical patients who have a SSI, NS + S (A x D)	D	3.6%	1,127,574 x 3.6% = 40,593	Russo et al. [17]
Proportion of surgical patients who don’t have a SSI, NS + S (A x E)	E	96.4%	1,127,574 x 96.4% = 1,086,981	Russo et al. [17]

AIHW = Australian Institute for Health & Welfare; SSI = surgical site infection; NDSHS = National Drug Strategy Household Survey; NS = non-smoking population; S = smoking population.

Smoking rates tend to be higher in surgical populations compared with the general population [31]. In the last 10 years, three Australian studies have reported smoking rates in orthopaedic trauma [26, 27] and acute fracture patients [28] between 19.6% and 32.8%. Older evidence suggested smoking rates in surgical patients could be as high as 38% [28]. Consequently, the analysis assumes an estimated surgical patient smoking rate of 23.9%, i.e. midway between the lowest and highest estimates, adjusting for trends in smoking rates over time consistent with the Australian smoking rate, i.e. 1.7% and 3.0% reduction between 2016–17 and 2015, and 2016–17 and 2011 respectively [32]. Additionally, uncertainty associated with this input into the analysis was incorporated using a probabilistic sensitivity analysis.

The numbers of smoking and non-smoking surgical patients experiencing a SSI were estimated by reverse engineering the odds ratio for SSIs reported by Sorensen and colleagues [10, 13] applied to the total number of surgical patients who experience a SSI from Table 1 (see Table 2).

**Table 2:** Estimated annual expected number of smoking and non-smoking surgical patients having a SSI in Australian public hospitals.

	SSI	No SSI	Total
Smoking surgical patients	14,386	255,105	269,490*
Non-smoking surgical patients	26,207	831,877	858,084*
Total	40,593*	1,086,981*	1,127,574*
Pooled odds ratio for SSI	1.79		

*numbers taken from Table 1; SSI = surgical site infection.

The total annual hospitalisation cost due to SSIs was estimated based on the annual number of excess HBDs associated with SSIs multiplied by the average cost per HBD (see Table 3). The number of excess HBDs associated with SSIs in Australia was taken from Graves et al. (2009) [25]. The average cost per HBD was calculated by dividing the most recently reported average cost per weighted episode of admitted acute care (2016–17; A$5,171) by the average length of stay for admitted acute patients (2.43) [29]. The weighting controls for differences in the complexity of care of each hospital admission relative to the other types of hospital admission [29]. More details on the weighting procedure are provided by the Australian Independent Hospital Pricing Authority [33]. All costs are reported in 2016 A$.

**Table 3:** Estimated annual expected number of surgical site infections, hospital bed-days and costs for smoking and non-smoking surgical patients in Australian public hospitals.

	Variable	Number (95% UI)	Source
Total number of smoking surgical patients having a SSI	A	14,386 (10,938, 18,604)	Table 2
Total number of non-smoking surgical patients having a SSI	B	26,207 (20,505, 32,619)	Table 2
Total number of surgical patients with a SSI (A + B)	C	14,386 + 26,207 = 40,593 (32,543, 50,239)	Table 2
Total number of excess HBDs for smoking surgical patients due to SSI (A x 2.51)	D	14,386 x 2.51 = 36,108 (17,369, 74,592)	Graves et al. (2009) [25]
Total number of excess HBDs for non-smoking surgical patients due to SSI (B x 2.51)	E	26,207 x 2.51 = 65,780 (31,969, 131,376)	Graves et al. (2009) [25]
Total excess HBDs for smoking and non-smoking surgical patients due to SSI (D + E)	F	36,108 + 65,780 = 101,888 (49,988, 200,822)	Calculated
Total hospitalisation costs for excess HBDs for smoking surgical patients (D x $2,128^#^)	G	$2,128 x 36,108 = $76,837,218 ($33,891,204, $166,167,787)	NHCDCR [29]
Total hospitalisation costs for excess HBDs for non-smoking surgical patients (E x $2,128^#^)	H	$2,128 x 65,780 = $139,977,890 ($61,948,864, $294,133,528)	NHCDCR [29]
Total hospitalisation costs for excess HBDs (F x $2,128^#^)	I	$2,128 x 101,888 = $216,815,107 ($93,524,091, $465,319,353)	NHCDCR [29]

^#^ the average cost per HBD calculated by dividing the average weighted episode of admitted acute care (A$5,171) by the average length of stay for admitted acute patients (2.43); NHCDCR = National Hospital Cost Data Collection Report; SSI = surgical site infections; PIF = potential impact fraction; HBD = hospital bed day; UI = uncertainty intervals.

### Impact analysis

In the base case analysis (analysis using the point estimates), potential annual cost savings from reduced SSIs, HBDs and associated hospitalisation costs were calculated assuming the estimated surgical smoking rate reduced from 23.9% to targets of 10% and 5% in 2016.

The potential impacts of reducing the smoking rate on reductions in SSIs were estimated using potential impact fractions (PIF). The PIF is the proportional change in SSI incidence due to changes in the smoking rate and was calculated according the following formula [34].


$$PIF=(p–p^{*})(RR–1)/p(RR–1)+1$$

Where p is the prevalence of smoking, p* is the counterfactual prevalence of smoking and RR is the relative risk of SSI in smoking surgical patients compared to non-smoking surgical patients. For example, if the smoking rate reduced from 23.9% to 5%, the potential impact fraction would be as follows (numbers taken from Tables 1 and 2):

PIF=(0.239–0.05)(1.748‐1)/0.239(1.748–1)+1=11.99%

Where the RR is calculated as (14,386/269,490) / (26,207/858,084), i.e. 1.748.

The number of surgical patients experiencing a SSI would decrease by 11.99% if the smoking rate was reduced from 23.9% to a target of 5%.

Generally, due to imperfect information on the inputs into the cost analysis, results are subject to uncertainty [8]. The impact of uncertainty around the parameters on the results is estimated using Monte Carlo simulation (1,000 iterations), executed with the Excel add-in software @RISK (Palisade Corporation), where values are drawn from probability distributions rather than being treated as a fixed value [35]. Uncertainty surrounding the average cost per episode of care, average length of stay, number of excess HBDs, odds ratio, total number of surgical procedures, surgical patient smoking rate and surgical site infection rate are included in the analysis. Distributions were selected according to modelling guidelines [36] (see S1 Appendix of Table 5). All calculations are presented in S2 Appendix of Calculations and S3 Appendix of Calculations_PSA. Please note, the probability sensitivity analysis will only run with the Excel add-in @RISK (Palisade Corporation).

## Results

According to the analysis, approximately 14,386 smokers (95% UI 10,938, 18,604) having a surgical procedure in Australian public hospitals in one year experienced a SSI (Table 2) with 36,108 extra HBDs (95% UI 17,369, 74,592) and $76.8M extra hospitalisation costs (95% UI $33.8M, $166.2M) (Table 3).

If the estimated surgical smoking rate reduced from 23.9% to 10%, the estimated potential hospital service cost savings are A$19.1M (95% UI $7.7M, $42.5M) with 8,985 HBDs saved (95% UI 4,094, 19,153) (Table 4). If the surgical smoking rate reduced further to 5%, then the estimated potential health system cost savings are A$26.0M (95% UI $10.8M, $57.0M) with 4,867 (95% UI 3,268, 6,867) SSIs prevented and 12,217 (95% UI 5,614, 25,642) HBDs saved.

**Table 4:** Estimated annual expected number of surgical site infections prevented and associated hospital bed-days and costs saved in Australian public hospitals resulting from reducing the surgical patient smoking rate from 23.9% to 10% or 5%.

	Variable	10% smoking rate	5% smoking rate	Source
Potential impact fraction	A	8.82%	11.99%	Zapata-Diomedi et al. (2018) [34]
Number of SSIs prevented (95% UI) (A x Row C, Table 3)	B	8.82% x 40,593 = 3,580 (2,312, 5,178)	11.99% x 40,593 = 4,867 (3,268, 6,867)	Table 3
Number of excess HBDs saved (95% UI) (B x 2.51)	C	3,580 x 2.51 = 8,985 (4,094, 19,153)	4,867 x 2.51 = 12,217 (5,614, 25,642)	Graves et al. (2009) [25]
Hospitalisation costs saved (95% UI) (C x $2,128^#^)	D	8,985 x $2,128 = $19,120,176 ($7,682,599, $42,515,662)	12,217 x $2,128 = $25,997,938 ($10,819,790, $56,960,886)	NHCDCR [29]

^#^ the average cost per HBD calculated by dividing the average weighted episode of admitted acute care (A$5,171) by the average length of stay for admitted acute patients; HBD = hospital bed-day; SSI = surgical site infection; UI = uncertainty intervals.

Estimates for each Australian state and territory are provided in the S4 Appendix of Table 6 for reaching the 5% smoking target. The largest estimated savings were predicted for New South Wales (A$5.0M, 95% UI $1.9M, $9.5M) and the greatest number of HBDs saved for Victoria (2,483, 95% UI 1,100, 5,076). Estimated savings across states and territories ranged widely from A$0.2M to A$3.6M and A$0.3M to A$5.0M for the 10% and 5% smoking rate targets, respectively (S2 Appendix of Calculations).

## Discussion

The present study estimated the effect of reducing the daily smoking rate amongst surgical patients in Australia on the expected number of SSIs and associated HBDs and hospitalisation costs. The findings suggest that reducing the estimated surgical smoking rate from 23.9% to 10% or 5% could result in annual hospitalisation cost savings of $19.1M (95% UI $7.7M, $42.5M) or $26.0M (95% UI $10.8M, $57.0M) respectively, by reducing hospital inpatient stays associated with SSIs. These are likely conservative estimates because smoking increases the risk of other post-operative complications associated with surgery, such as respiratory failure, lung infections and pneumonia [16, 37] and the costs of treating SSIs are not considered in the estimates. Additionally, SSIs can also lead to readmissions [38]. Reducing the surgical smoking rate would likely also reduce readmissions and associated HBDs with additional benefits realised. Cost estimates are based on the perspective of healthcare providers and do not include cost impacts on patients, such as out of pocket expenses and time costs, nor do the estimates include costs to society, such as productivity loss.

The estimated expected health system cost savings presented in this analysis are based on overall surgical population reductions in smoking. Providing salient advice to quit when patients are likely to be highly motivated to reduce surgery complications could yield even greater reductions in smoking prevalence [39–41]. The potential additional cost savings would likely be offset, to some extent, by costs to provide TDT principally in the provision of smoking cessation pharmacotherapy and the time taken to refer patients to services providing multi-session behavioural interventions, such as quitlines (which are funded by state and territory governments in Australia). Embedding TDT in routine care for hospitalised patients is clinically effective and highly cost-effective with incremental cost-effectiveness ratios well below the estimated Australian Government threshold for judging value for money [3, 37, 42, 43].

Recent reductions in federal hospital funding means administrators are facing escalating financial pressures while trying to achieve demanding waiting time targets [44, 45]. The analysis suggests approximately 36,108 (95% UI 17,369, 74,592) *extra* HBDs are being utilised every year due to increased SSI rates among people who smoke undergoing a surgical procedure. Embedding best practice TDT in hospitals could increase annual bed-day capacity by as much as 12,217 days (95% UI 5,614, 25,642) if a 5% smoking rate was achieved.

The annual number of SSIs prevented, HBDs and hospitalisation costs saved vary substantially between states and territories in Australia (see S4 Appendix of Table 6). These differences are driven by variations in the total number of surgical procedures, smoking rates, average length of hospital stay and average cost per acute separation.

Whilst the findings from this analysis are applicable to the Australian setting, the methods presented can be applied to jurisdictions with similar healthcare systems funded predominantly by public insurance, such as Canada, Belgium and France [46], available data permitting.

### Limitations

Ideally, cost estimates should be derived from the most recent, robust, prospectively collected epidemiological and cost data for surgical procedures in Australian public hospitals surgical smoking rates and SSIs. However, empirical data on 2016–17 acute public hospital admissions were used to inform key steps in the calculations because of the absence of evidence on the average cost per surgical admission and average length of stay for admitted acute surgical patients. Data on surgical smoking rates in Australia is limited. The estimates are predicated on a 23.9% smoking rate which may not accurately reflect the 2016–17 surgical patient smoking rates. However, wide uncertainty intervals (17.9%, 29.8%) are included in the probabilistic sensitivity analysis to account for this parameter uncertainty in the final estimates [36] and recent evidence suggests the assumed surgical patient smoking rate is plausible [31]. Unfortunately, recent estimates on the number of excess HBDs associated with SSIs in Australia are scarce. Consequently, this analysis applies an estimate (2.51 excess HBDs) derived from surgical patients admitted to three Australian hospitals in 2004 which may not accurately reflect acute care in 2016–17 given changes in healthcare provision. Evidence suggests the number of excess HBDs associated with a SSI varies according to surgical procedure [15]. However, the analysis is not stratified by type of surgery. Rather an average number of excess HBDs is applied derived from data collected across 23 clinical specialities [25]. The analysis does not account for possible differences in excess HBDs by smoking status. Given smokers tend to use more HBDs than non-smokers generally [47], the estimated number of HBDs and healthcare costs saved from reducing the surgical smoking rate to 5 or 10% is likely to be even greater. Of note, a recent systematic review on the impact of SSIs reported much higher excess HBDs (median 18, range 2–55) across a variety of surgical specialities in six European countries [15]. The results represent the best estimate of a potential effect at the time of analysis in the absence of stronger direct evidence [8].

Finally, estimates are predicated on *ceteris parabis*, i.e. the effect of reducing the smoking rate on SSIs, excess HBDs and hospitalisation costs, assuming all other variables remain the same. A modelled economic evaluation synthesising the best available evidence is needed to more accurately evaluate the costs and consequences of providing preoperative smoking cessation interventions on postoperative complications.

## Conclusions

Providing best practice TDT as part of routine care prior to surgery could substantially improve hospital bed utilisation and surgery outcomes. The economic benefits are highly likely to exceed the costs of providing TDT, although a more comprehensive modelled economic evaluation is needed to confirm the findings.

## Supporting information

S1 Appendix Table 5 Parameters and distributions used in the calculations. (DOCX)

S2 Appendix Calculations. (XLSX)

S3 Appendix Calculations_PSA. (XLSX)

S4 Appendix Table 6 Estimated annual expected number of surgical site infections prevented and associated hospital bed-days and costs saved in Australian state and territory public hospitals if a 5% smoking target is achieved. (DOCX)
