# Supplementary material for: What is the likely impact on surgical site infections in Australian hospitals if smoking rates are reduced? A cost analysis
Source: PLoS One. 2021 Aug 25;16(8):e0256424. doi: 10.1371/journal.pone.0256424 (PMC8386862; doi:10.1371/journal.pone.0256424)
Supplement: S1 Appendix — (DOCX) [file pone.0256424.s001.docx]

**Table 5 Parameters and distributions used in the calculations**

| **Parameter** | **Baseline estimate** | **Range** | **Distribution** | **SD** | **Source** |
| --- | --- | --- | --- | --- | --- |
| Average cost per episode of care | $5,171 | $3,972 - $6,475 | Gamma | $762 | NHCDCR (2019) [29] |
| Average LOS | 2.43 | 1.97-2.88 | Gamma | 0.28 | NHCDCR (2019) [29] |
| Number of excess HBDs | 2.51 | 1.27-4.92 | Gamma | 1.22 | Graves et al (2009) [25] |
| Total number of surgical procedures | 1,127,574 | 1,014,817 – 1,240,331 | Pert | 42,618 | AIHW (2018) [24] |
| Smoking rate for surgical patients | 0.239 | 0.179-0.298 | Pert | 0.02 | McCrabb et al (2017) [26]  McCrabb et al (2019) [27]  Neptune et al (2014) [28] |
| SSI rate | 0.036 | 0.029-0.044 | Pert | 0.01 | Russo et al (2019) [17] |
| OR for surgical patients who smoke experiencing a SSI versus non-smokers | 1.79 | 1.57-2.04 | Logistic | 0.145 | Sorensen at al (2012) [13] |

HBD = hospital bed day; LOS = length of stay; OR = odds ratio; SSI = surgical site infection rate
