# Supplementary material for: What is the likely impact on surgical site infections in Australian hospitals if smoking rates are reduced? A cost analysis
Source: PLoS One. 2021 Aug 25;16(8):e0256424. doi: 10.1371/journal.pone.0256424 (PMC8386862; doi:10.1371/journal.pone.0256424)
Supplement: S4 Appendix — (DOCX) [file pone.0256424.s004.docx]

| **State/ territory** | **Smoking rate** | **Number of SSIs prevented (95% UI)** | **Number of HBDs saved (95% UI)** | **Hospitalisation costs saved (95% UI)** |
| --- | --- | --- | --- | --- |
| NSW | 22.4% | 958 (708, 1,422) | 2,404 (1,186, 5,598) | $4,972,438 ($1,892,432, $9,517,192) |
| VIC | 23.0% | 989 (679, 1.329) | 2,483 (1,100, 5,076) | $4,374,977 ($2,226,451, $10,807,775) |
| QLD | 28.4% | 711 (487, 952) | 1,784 (821, 3,760) | $4,232,734 ($1,751,666, $9,336,006) |
| WA | 22.8% | 358 (248, 491) | 899 (418, 1,889) | $2,609,104 ($1,114,417, $5,985,943) |
| SA | 21.3% | 245 (168, 337) | 614 (277, 1,273) | $1,264,768 ($531, 639, $2,768,240) |
| TAS | 31.6% | 94 (66, 126) | 237 (106, 499) | $462,903 ($201,467, $1,062,215) |
| ACT | 18.5% | 55 (36, 74) | 137 (63, 277) | $299,141 ($128,305, $671,830) |
| NT | 34.5% | 52 (37, 70) | 131 (61, 278) | $263,377 ($113,922, $608,981) |
| Australia | 23.9% | 4,867 (3,268, 6,867) | 12,217 (5,614, 25,642) | $25,997,938 ($10,819,790, $56,960,886) |

ACT = Australian Capital Territory; HBD = hospital bed-day; QLD = Queensland; NSW = New South Wales; NT = Northern Territory; SA = South Australia; SSI = surgical site infection; TAS = Tasmania; UI = Uncertainty Intervals; VIC = Victoria; WA = Western Australia;
